# Optimizing search strategies to identify randomized controlled trials in MEDLINE

**DOI:** 10.1186/1471-2288-6-23

**Published:** 2006-05-09

**Authors:** Li Zhang, Isola Ajiferuke, Margaret Sampson

**Affiliations:** 1 Natural Sciences Library, University of Saskatchewan, Saskatoon, Canada; 2 Faculty of Information and Media Studies, University of Western Ontario, London, Canada; 3 Chalmers Research Group, Children's Hospital of Eastern Ontario Research Institute, Ottawa, Canada

## Abstract

**Background:**

The Cochrane Highly Sensitive Search Strategy (HSSS), which contains three phases, is widely used to identify Randomized Controlled Trials (RCTs) in MEDLINE. Lefebvre and Clarke suggest that reviewers might consider using four revisions of the HSSS. The objective of this study is to validate these four revisions: combining the free text terms *volunteer, crossover, versus*, and the Medical Subject Heading *CROSS-OVER STUDIES *with the top two phases of the HSSS, respectively.

**Methods:**

We replicated the subject search for 61 Cochrane reviews. The included studies of each review that were indexed in MEDLINE were pooled together by review and then combined with the subject search and each of the four proposed search strategies, the top two phases of the HSSS, and all three phases of the HSSS. These retrievals were used to calculate the sensitivity and precision of each of the six search strategies for each review.

**Results:**

Across the 61 reviews, the search term *versus *combined with the top two phases of the HSSS was able to find 3 more included studies than the top two phases of the HSSS alone, or in combination with any of the other proposed search terms, but at the expense of missing 56 relevant articles that would be found if all three phases of the HSSS were used. The estimated time needed to finish a review is 1086 hours for all three phases of the HSSS, 823 hours for the strategy *versus*, 818 hours for the first two phases of the HSSS or any of the other three proposed strategies.

**Conclusion:**

This study shows that compared to the first two phases of the HSSS, adding the term *versus *to the top two phases of the HSSS balances the sensitivity and precision in the reviews studied here to some extent but the differences are very small. It is well known that missing relevant studies may result in bias in systematic reviews. Reviewers need to weigh the trade-offs when selecting the search strategies for identifying RCTs in MEDLINE.

## Background

The new century has seen a significant proliferation of systematic reviews, and they have become one of the key tools for the evidence-based medicine movement. A quality systematic review involves a comprehensive search for relevant studies on a specific topic, and those identified are then assessed and synthesized according to a predetermined and explicit method [1]. Although the studies evaluated in systematic reviews can be any kind of research [2], reviewers of the effects of health care interventions tend to base their reviews on Randomized Controlled Trials (RCTs), when possible, as they are one of the most rigorous study designs [3]. Comprehensive searching, when conducting systematic reviews of RCTs, is considered a standard practice [4]. It has long been assumed that information specialists should use highly sensitive search strategies to identify potentially relevant primary studies for systematic reviews. Sensitivity and precision are two parameters to evaluate the performances of a search strategy. Sensitivity is defined as the proportion of relevant studies retrieved, while precision is the proportion of retrieved studies that are relevant. An ideal search strategy would have both high sensitivity and high precision, which means most of the available relevant items in a database are retrieved by the search strategy and most of the items retrieved by the search strategy are relevant. However, sensitivity and precision are inversely related. The higher the sensitivity, the lower the precision [5]. This means that when a highly sensitive search strategy is used, many irrelevant studies are retrieved, thus increasing the workload for the researchers conducting the systematic research. In practice, reviewers, restricted by time and cost, must strive to identify the maximum number of eligible trials, hoping that the studies included in the review will be a representative sample of all eligible trials [6]. The overall time and cost of doing a systematic review is dependent on the size of initial bibliographic retrieval [7]. Thus, fine-tuning this initial step in the review process can yield great efficiencies.

The MEDLINE database, created and maintained by the United States National Library of Medicine, is the most widely-used database in medicine and other health science fields. It includes 15 million citations dating back to the mid-1960's [8]. The Highly Sensitive Search Strategy (HSSS) [9] is a standard search strategy recommended by the Cochrane Collaboration to identify RCTs in the MEDLINE database. It was developed in the early 1990's and contains three phases (See Additional file 1). While agreeing that the top two phases of the HSSS should always be used to identify RCTs in MEDLINE, a pilot study by the U.K. Cochrane Centre in 1994 concluded that the terms of the third phase were too broad to warrant their inclusion in the MEDLINE Retagging Project [10,11]. Another study found that the search for RCTs on hypertension was sufficiently sensitive only when all three phases of the search strategy were used [12]. As Lefebvre and Clarke [11] suggest, it would not be worth applying all three phases but individual reviewers might consider it worth combining the top two phases with individual terms, such as the free-text terms *volunteer*, *crossover *and *versus*, and the Medical Subject Heading (MeSH) *CROSS-OVER STUDIES *which was introduced after the search strategy was devised.

A few recent studies [13,14] have explored different search strategies to identify RCTs in MEDLINE. Haynes and colleagues [13] developed separate strategies for different purposes: strategies with high sensitivity for comprehensive searching and strategies with high precision for more focused searching. Robinson and Dickersin [14] tested a revised search strategy of all three phases of the HSSS for OVID MEDLINE and PubMed. This strategy has a better performance than the original HSSS. Because an increasing number of systematic reviews have to be completed within tight budgets and timelines, it is sometimes necessary to strike a balance between comprehensiveness and precision. Several proposals to refine that balance by making minor modifications to the first two phases of the HSSS were put forth by Lefebvre and Clarke in 2001 [11]. A comprehensive literature search reveals that there are no published data that evaluate the performances of the four search strategies proposed. Because balancing the initial retrieval size greatly improves the efficiency of a systematic review, we tested the performances of these four proposed revisions of the HSSS: combining the top two phases of HSSS with the free-text terms *volunteer*, *crossover*, *versus*, and the MeSH term *CROSS-OVER STUDIES*, respectively [11].

## Methods

### Selection of systematic reviews

Systematic reviews, which might have used the HSSS to identify RCTs, were selected from the Cochrane Database of Systematic Reviews (CDSR), OVID interface (1^st ^Quarter 2003) using the following search strategy:

(hsss.tw.) or (highly sensitive search.tw.)

These systematic reviews were then screened using three eligibility criteria. To be selected, each systematic review had to use at least one phase of the HSSS, report the citations for included and excluded studies, and indicate if primary studies were either RCTs or quasi RCTs.

### Finding the index status of each included study

We did a known-item search for the included studies of each systematic review that met the three inclusion criteria in OVID MEDLINE (1966 – 2003) to determine whether they were indexed in MEDLINE or not. The bibliographic records of the included studies that were indexed in MEDLINE were aggregated together by review using the Boolean operator "or". Each known-item search strategy was saved in OVID MEDLINE. We recorded the number of included studies that were indexed in MEDLINE for each systematic review. This was used to calculate sensitivity for each review. One sample of the known-item search strategy is listed in Additional file 2.

### Test search strategies

Each of the four candidate terms the free-text terms *volunteer, crossover, versus *and MeSH term *CROSS-OVER STUDIES *was combined with the first two phases of the HSSS to create four test strategies with the Boolean operator "or" (See Additional file 1. The search strategies are hereafter abbreviated as SS_volunteer_, SS_crossover_, SS_versus_, SS_CROSS-OVERSTUDIES_, respectively).

### Sensitivity

**Figure 1:**
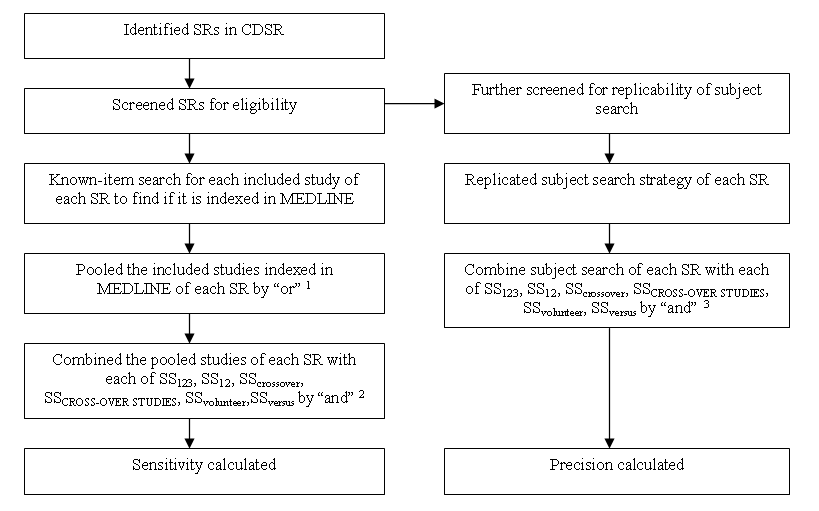
Steps of how sensitivity and precision were calculated. Note 1. The number of the pooled studies of each SR is the denominator of the sensitivity for each SR. Note 2. The number of items retrieved in this step is the nominator of the sensitivity and the precision for each SR. Note 3. The number of items retrieved in this step is the initial search output (articles needed to screen) of a SR, which is also the denominator of the precision. SR = systematic review

**Figure 2:**
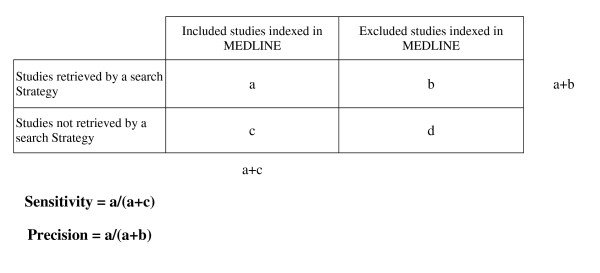
Formula for calculating sensitivity and precision.

For each of the systematic reviews that met the three inclusion criteria, we combined the pooled included studies indexed in MEDLINE and each of the four test search strategies, all three phases of the HSSS (hereafter abbreviated as SS_123_), and the top two phases of the HSSS (hereafter abbreviated as SS_12_) by using Boolean operator "and" in OVID MEDLINE (1966-February 2004) to find out how many included studies indexed in MEDLINE were retrieved by each search strategy (See Figure 1). We recorded the numbers of included trials indexed in MEDLINE that could be retrieved by each of SS_123_, SS_12_, SS_crossover_, SS_CROSS-OVERSTUDIES_, SS_volunteer_, and SS_versus_. Based on these data, the sensitivity of each strategy for each review was calculated (See Figure 2). More specifically, the sensitivity for each review is defined as:

**Sensitivity **= No. of included studies indexed in Medline that were retrieved by a search strategyNo. of included studies indexed in Medline
 MathType@MTEF@5@5@+=feaafiart1ev1aaatCvAUfKttLearuWrP9MDH5MBPbIqV92AaeXatLxBI9gBaebbnrfifHhDYfgasaacH8akY=wiFfYdH8Gipec8Eeeu0xXdbba9frFj0=OqFfea0dXdd9vqai=hGuQ8kuc9pgc9s8qqaq=dirpe0xb9q8qiLsFr0=vr0=vr0dc8meaabaqaciaacaGaaeqabaqabeGadaaakeaadaWcaaqaaiabb6eaojabb+gaVjabb6caUiabbccaGiabb+gaVjabbAgaMjabbccaGiabbMgaPjabb6gaUjabbogaJjabbYgaSjabbwha1jabbsgaKjabbwgaLjabbsgaKjabbccaGiabbohaZjabbsha0jabbwha1jabbsgaKjabbMgaPjabbwgaLjabbohaZjabbccaGiabbMgaPjabb6gaUjabbsgaKjabbwgaLjabbIha4jabbwgaLjabbsgaKjabbccaGiabbMgaPjabb6gaUjabbccaGiabb2eanjabbwgaLjabbsgaKjabbYgaSjabbMgaPjabb6gaUjabbwgaLjabbccaGiabbsha0jabbIgaOjabbggaHjabbsha0jabbccaGiabbEha3jabbwgaLjabbkhaYjabbwgaLjabbccaGiabbkhaYjabbwgaLjabbsha0jabbkhaYjabbMgaPjabbwgaLjabbAha2jabbwgaLjabbsgaKjabbccaGiabbkgaIjabbMha5jabbccaGiabbggaHjabbccaGiabbohaZjabbwgaLjabbggaHjabbkhaYjabbogaJjabbIgaOjabbccaGiabbohaZjabbsha0jabbkhaYjabbggaHjabbsha0jabbwgaLjabbEgaNjabbMha5bqaaiabb6eaojabb+gaVjabb6caUiabbccaGiabb+gaVjabbAgaMjabbccaGiabbMgaPjabb6gaUjabbogaJjabbYgaSjabbwha1jabbsgaKjabbwgaLjabbsgaKjabbccaGiabbohaZjabbsha0jabbwha1jabbsgaKjabbMgaPjabbwgaLjabbohaZjabbccaGiabbMgaPjabb6gaUjabbsgaKjabbwgaLjabbIha4jabbwgaLjabbsgaKjabbccaGiabbMgaPjabb6gaUjabbccaGiabb2eanjabbwgaLjabbsgaKjabbYgaSjabbMgaPjabb6gaUjabbwgaLbaaaaa@CA09@

### Precision

Each systematic review that met the three eligibility criteria was further screened to determine if the subject search was presented in enough detail to permit replication. For the reviews with a detailed description of the subject search, we replicated the search strategy of the specific topic (e.g., search strategy to find "hormone"-related studies). We assumed that the subject search presented in each review was a comprehensive search for identifying the subject- related studies. The subject search strategy was combined with each of SS_123_, SS_12_, SS_crossover_, SS_CROSS-OVERSTUDIES_, SS_volunteer_, and SS_versus _using the Boolean operator "and" (See Additional file 1). If the subject search presented in the Cochrane reviews was conducted in MEDLINE interfaces other than OVID (e.g., SilverPlatter), the search was converted into OVID syntax. This gave us the size of the initial search retrieval. We recorded the number of the initial retrieval for each review, which was used to calculate the precision (See Figure 1). Based on these data, the precision of each search strategy was calculated for each review (See Figure 2). The precision of each search strategy for each review is defined as:

**Precision **= No. of included studies indexed in MEDLINE that were retrieved by a search strategyNo. of studies initially retrieved by the search strategy
 MathType@MTEF@5@5@+=feaafiart1ev1aaatCvAUfKttLearuWrP9MDH5MBPbIqV92AaeXatLxBI9gBaebbnrfifHhDYfgasaacH8akY=wiFfYdH8Gipec8Eeeu0xXdbba9frFj0=OqFfea0dXdd9vqai=hGuQ8kuc9pgc9s8qqaq=dirpe0xb9q8qiLsFr0=vr0=vr0dc8meaabaqaciaacaGaaeqabaqabeGadaaakeaadaWcaaqaaiabb6eaojabb+gaVjabb6caUiabbccaGiabb+gaVjabbAgaMjabbccaGiabbMgaPjabb6gaUjabbogaJjabbYgaSjabbwha1jabbsgaKjabbwgaLjabbsgaKjabbccaGiabbohaZjabbsha0jabbwha1jabbsgaKjabbMgaPjabbwgaLjabbohaZjabbccaGiabbMgaPjabb6gaUjabbsgaKjabbwgaLjabbIha4jabbwgaLjabbsgaKjabbccaGiabbMgaPjabb6gaUjabbccaGiabb2eanjabbweafjabbseaejabbYeamjabbMeajjabb6eaojabbweafjabbccaGiabbsha0jabbIgaOjabbggaHjabbsha0jabbccaGiabbEha3jabbwgaLjabbkhaYjabbwgaLjabbccaGiabbkhaYjabbwgaLjabbsha0jabbkhaYjabbMgaPjabbwgaLjabbAha2jabbwgaLjabbsgaKjabbccaGiabbkgaIjabbMha5jabbccaGiabbggaHjabbccaGiabbohaZjabbwgaLjabbggaHjabbkhaYjabbogaJjabbIgaOjabbccaGiabbohaZjabbsha0jabbkhaYjabbggaHjabbsha0jabbwgaLjabbEgaNjabbMha5bqaaiabb6eaojabb+gaVjabb6caUiabbccaGiabb+gaVjabbAgaMjabbccaGiabbohaZjabbsha0jabbwha1jabbsgaKjabbMgaPjabbwgaLjabbohaZjabbccaGiabbMgaPjabb6gaUjabbMgaPjabbsha0jabbMgaPjabbggaHjabbYgaSjabbYgaSjabbMha5jabbccaGiabbkhaYjabbwgaLjabbsha0jabbkhaYjabbMgaPjabbwgaLjabbAha2jabbwgaLjabbsgaKjabbccaGiabbkgaIjabbMha5jabbccaGiabbsha0jabbIgaOjabbwgaLjabbccaGiabbohaZjabbwgaLjabbggaHjabbkhaYjabbogaJjabbIgaOjabbccaGiabbohaZjabbsha0jabbkhaYjabbggaHjabbsha0jabbwgaLjabbEgaNjabbMha5baaaaa@DCB2@

## Results

**Figure 3:**
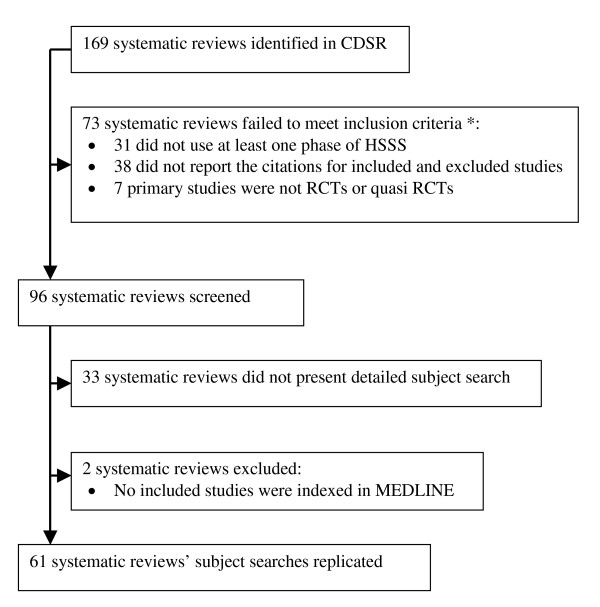
Flow of systematic reviews through the phase of screening and eligibility evaluation. * For some reviews, more than one exclusion criteria was noted, therefore numbers do not add up to 73.

We identified 169 systematic reviews from CDSR, which represented about 10% of the reviews published in the database in 2003. Of the 169 systematic reviews, 96 reviews met the three inclusion criteria. Of the 96 reviews, 61 reviews (63.54%) presented detailed subject search to allow us to replicate their subject search (See Additional file 3); 33 (34.38%) systematic reviews did not list detailed subject search strategies; in 2 (2.08%) reviews, none of the included studies was indexed in MEDLINE (See Figure 3). The median number of included studies per review is 12 studies. The 61 reviews were done by different Review Groups, mainly Musculoskeletal Injuries, Eyes and Vision, Renal, Prostatic Diseases and Urologic Cancers, Back, Upper Gastrointestinal and Pancreatic Diseases, and Skin. The review group that listed the largest number of detailed search strategies is Musculoskeletal Injuries Review Group (13 reviews, 21.3%). Characteristics of included studies are presented in Table 1.

**Table 1:** Characteristics of included studies.

	n (total = 61)	%
Year of publication or substantive update		
Median	2001	
Interquartile range	1999–2001	
Focus of the review		
Treatment	52	85.2
Prevention	7	11.5
Diagnosis	1	1.6
Other	1	1.6
Study designs included		
RCT only	33	54.1
RCT and quasiRCT	24	39.3
RCT and other controlled trials	4	6.6
Number of included studies per review		
Median	12	
Interquartile range	5.5–19.5	
Review Group		
Musculoskeletal Injuries	13	21.3
Eyes and Vision	11	18.0
Renal	6	9.8
Prostatic Diseases and Urologic Cancers	5	8.2
Back	4	6.6
Upper Gastrointestinal and Pancreatic Diseases	3	4.9
Skin	3	4.9
Other	16	30.0

We were able to calculate the sensitivity for 94 reviews. The overall sensitivity of the four proposed search strategies, SS_12_, and SS_123 _is very high, with the same median of 100%. For SS_crossover_, SS_CROSS-OVERSTUDIES_, SS_volunteer_, SS_versus_, and SS_12_, 52% of the reviews achieved a perfect sensitivity (100%). For SS_123_, 70% of the reviews achieved a perfect sensitivity. A closer examination of the data found that, across the 94 reviews, SS_versus _was able to find 3 more relevant articles than SS_crossover_, SS_CROSS-OVERSTUDIES_, SS_volunteer_, or SS_12_, but SS_123 _found 56 more relevant articles than SS_versus_. There is no obvious difference between the sensitivities of the 61 reviews which listed their detailed subject specific search strategies and those of the 33 reviews which did not list their search strategies (The medians of the sensitivities for each search strategy for both categories are all 100%). The sensitivity of the four test search strategy, SS_12_, and SS_123 _are presented in Table 2.

**Table 2:** Sensitivity of each search strategy.

Search strategy	Median (%)	Interquartile range (%)	Mean rank (Friedman test)	Reviews with perfect sensitivity (%)	Relevant items missed (across 94 reviews)
SS_123_	100.00	4.17	4.24	70.21	49
SS_12_	100.00	12.15	3.34	52	108
SS_crossover_	100.00	12.15	3.34	52	108
SS_CROSS-OVERSTUDIES_	100.00	12.15	3.34	52	108
SS_volunteer_	100.00	12.15	3.34	52	108
SS_versus_	100.00	12.15	3.41	52	105

χ^2 ^= 131.667, p < .01

We were able to calculate the precision for the 61 reviews presented a detailed subject search. The precision of the six search strategies can be found in Table 3. The precision of the four proposed search strategies and the top two phases of HSSS (Median range: 1.48% – 1.68%) was much higher than that of the all three phases of HSSS (median 0.54%). The size of initial retrieval of each search strategy is shown in Table 4. The medians of initial retrieval of the four proposed search strategies and SS_12 _for each review range from 408–430 studies, and the median of SS_123 _is 1636 studies. The median of initial retrieval of SS_versus _is about 1/4 of that of SS_123_, which means the number of articles needed to read by reviewers would be reduced significantly if SS_versus _instead of SS_123 _was used to identify RCTs. When SS_123 _was used, 36 reviews (59.02%) had a very low precision (less than 1%); when SS_12_, SS_crossover_, SS_CROSS-OVERSTUDIES_, or SS_volunteer _was used, 21 reviews (34.43%) had a precision less than 1%; When SS_versus _was used, 22 reviews (36.07%) had a precision less than 1%. Table 4 also shows the Article Read Ratio (ARR), which is defined as the median of articles initially retrieved divided by the median of included studies retrieved. The ARR of SS_123 _(182) is significantly higher than that of SS_versus _(54), and the ARRs of SS_12_, SS_crossover_, SS_CROSS-OVERSTUDIES_, and SS_volunteer _are the same (51). We calculated the estimated time to finish a review for each search strategy based on the regression equation developed by Allen and Olkin [7]: time = 721 + 0.243 *x *- 0.0000123*x*^2^, where *x *denotes the number of articles initially retrieved, as shown in Table 4. The time needed to finish a review is 1086 hours for SS_123_, 823 hours for SS_versus_, 818 hours for SS_12_, SS_crossover_, SS_CROSS-OVERSTUDIES_, or SS_volunteer_.

**Table 3:** Precision of each search strategy.

Search strategy	Median (%)	Interquartile range (%)	Mean rank (Friedman test)	Reviews with precision < 1% (%)	Total retrieval size (across 61 reviews)
SS_123_	0.54	1.87	1.02	59.02	508625
SS_12_	1.68	5.55	5.17	34.43	151691
SS_crossover_	1.68	5.54	4.24	34.43	152662
SS_CROSS-OVERSTUDIES_	1.68	5.55	4.77	34.43	151844
SS_volunteer_	1.68	5.51	3.52	34.43	152880
SS_versus_	1.48	5.04	2.29	36.07	171032

χ^2 ^= 258.634, p < .01

**Table 4:** Size of initial retrieval per review.

Search strategy	Size of initial retrieved articles Median (interquartile range)	Size of included studies retrieved Median (interquartile range)	Article Read Ratio^1^	Estimated time to finish a review (hours)^2^
SS_123_	1636 (4042)	9 (14)	182	1086
SS_12_	408 (1210)	8 (12)	51	818
SS_crossover_	408 (1216)	8 (12)	51	818
SS_CROSS-OVERSTUDIES_	408 (1211)	8 (12)	51	818
SS_volunteer_	408 (1235)	8 (12)	51	818
SS_versus_	430 (1361)	8 (12)	54	823

Note 1. Article Read Ratio = Median of Initial Retrieved Articles/Median of Included Studies Retrieved

Note 2. Estimated time to finish a review = 721 + 0.243x - 0.0000123x2, where x denotes the median of size of initial retrieved articles showed in Column 2 of this table.

## Discussion

Searching bibliographic databases to identify relevant studies is one of the most important steps of a systematic review [6]. All the systematic reviews identified in this research searched MEDLINE; therefore, developing an effective MEDLINE search strategy is an integral component of a comprehensive search plan [14]. We validated four previously proposed variants of the HSSS by testing their retrieval characteristics. Across the 61 reviews, the performance of SS_crossover_, SS_CROSS-OVERSTUDIES_, and SS_volunteer _are the same as SS_12_. SS_123 _found 56 more relevant articles than SS_versus_, and SS_versus _found 3 more relevant articles than SS_12_, SS_crossover_, SS_CROSS-OVERSTUDIES_, or SS_volunteer_. The number of articles needed to read per review when SS_versus _is used is about 1/4 of that when SS_123 _is used, and the estimated time to finish a review for SS_123 _is 32% higher than that for SS_versus_. On the other hand, the number of articles needed to read when SS_versus _is used is only 5% (22 additional articles) more than that when SS_12 _is used, and the estimated time to finish a review for SS_versus _is 0.6% (5 hours) more than that for SS_12_. The result shows that, compared to SS_123_, SS_versus _will reduce the number of articles needed to read significantly, thus reducing the reviewers' work in assessing citations for eligibility and the total time to complete a review, while still maintaining a workload comparable to SS_12 _but a slightly better sensitivity than SS_12_. Although the other three proposed search strategies also have a lower initial retrieval size than SS_123_, their sensitivity is the same as SS_12_.

A comprehensive search is considered one of the key factors that distinguish a systematic review from a narrative review, and it is well-known that missing relevant studies will possibly result in bias for systematic reviews. This study confirms that SS_123 _will miss fewer relevant studies than SS_12_, and the four variants recommended by Lefebvre and Clarke, including SS_versus_. Because timelines and financial costs are usually a concern to most reviewers, they must decide whether these benefits justify the extra costs for much broader screening of the initial retrievals and the longer time needed to complete a review.

The comprehensiveness of systematic review searches not only depends on search filters but also on the varieties of databases searched. The Cochrane Central Register of Controlled Trials (CENTRAL) is the most comprehensive database of controlled trials. It is hoped that CENTRAL can serve as an all-inclusive source of controlled trials. When searching this database, reviewers need only to develop subject search strategies, thus avoiding the problem of selecting search filters. Each Collaborative Review Group (CRG) also develops a subject specialized register of trials to ensure that reviewers within the CRG have access to the maximum number of studies to their topic. All the reviews screened in this study indicated that they searched CENTRAL and/or the specialized CRG registers. The Cochrane Collaboration has gone to great effort to enhance the comprehensiveness of CENTRAL. Therefore, if reviewers do decide to use the first two phases of the HSSS and plan to search CENTRAL and/or the specialized CRG registers as well, they may consider using SS_versus _because it maintains a workload comparable to SS_12 _but a slightly better sensitivity. If reviewers do not have access to CENTRAL or the specialized registers, we suggest that they still use all three phases of the HSSS to maintain the quality of systematic reviews.

Since the first publication of the Highly Sensitive Search Strategy [9] few studies have examined the performance of the HSSS and its variations in general. Robinson and Dickersin [14] in their study testing a revision of the HSSS, concluded that adding the search terms "(latin adj square).tw." and "CROSS-OVER STUDIES" in all three phases of the HSSS would retrieve more controlled trials. Haynes and colleagues [13] recently developed a search strategy to identify RCTs in the MEDLINE that has a sensitivity of 99.3%. Across the medical information science field, although an increasing number of studies [15-22] have been done to test various search strategies to find RCTs in subject-specific areas, all of them tested the results on the RCT level. That is, their focus was determining how many studies identified by a search strategy were actually RCTs. Our study applied a different approach to test the performances of the search strategies. Because we tested how many studies included in a systematic review could be found through the four test strategies and the HSSS, our study is the first one that calculates the sensitivity and the precision of the search strategies at the systematic review level. Jenkins [23] states that the ultimate test of a search filter is to find out how well it performs in the real situation. We replicated the search strategies used in real systematic reviews, therefore, our method and results may have more practical significance for those conducting systematic reviews because they provide quantified data, including initial retrieval size and time needed to finish a review, to describe the cost-effectiveness of each search strategy. Systematic reviews should use evidence-based methods, and the validation of search filters is important in that context. Our contribution is to provide methodological rigor to previous non-validated recommendations. Thus reviewers can make informed decision based on this evidence.

The strength of a systematic review over any other kind of review is that it provides readers with an approach to replicate it [1]. The Quality of Reporting of Meta-analyses (QUOROM) statement suggests that a high-quality systematic review should explicitly describe all search strategies used to identify relevant studies [24]. The Cochrane Collaboration first recommended that reviewers report the full search strategies in the additional tables section of the Cochrane systematic review reports in 2002 [25], and the recent Cochrane Handbook for Systematic Reviews of Interventions clearly indicates that reviewers should describe their search strategies in sufficient detail so that the process could be replicated [3]. When screening the 96 Cochrane reviews, we found that 33 (34.38%) reviews did not describe their detailed search strategies, thus we could not replicate their search strategies. Of the 33 reviews, although a few listed their search strategies, they were not accurate. Common problems were that non-MeSH terms were listed as MeSH terms, and truncation was not used correctly. The 33 reviews that did not list the full search strategies all referred to the specific CRG search strategies with a hyperlink to individual CRG websites. But when browsing these websites, we did not find the search strategies. In order to improve the quality of reporting of systematic reviews, investigators should report their exact search strategies to allow readers to judge the breadth and depth of the search. If journals publishing systematic reviews do not have space for authors to list their full search strategies, we suggest that authors indicate that the search strategy is available upon request. Further, we suggest that the group search strategies for each CRG be documented on their websites. Our results mirror the findings of a recent study that assessed the quality of reporting of systematic reviews published in both CDSR and journals in pediatric complementary and alternative medicine (CAM) [26]. The study found that half of the CAM reviews reported the search terms used, but that very few (8.5%) actually listed the search strings. Our research indicates that the reporting quality of literature searches for systematic reviews has been improving but is still less than optimal.

Our study has some limitations. First, we tested only how many included studies could be retrieved by each of the six search strategies, not whether the 56 relevant studies missed by SS_versus _would change the outcomes of the 61 reviews. Therefore, we can not judge whether skipping Phase Three of the HSSS would result in bias in systematic reviews. Second, an effective search strategy for systematic reviews usually includes two parts: a subject search and a search filter, both of which are integral to the search strategy. In our study, we replicated the subject search for each systematic review, but the quality of these subject searches is unknown. There is a possibility that some relevant studies could be retrieved if the subject search were more comprehensive. Therefore, the sensitivity of the test search strategies could be lower, and precision higher than those we presented if the subject search were truly comprehensive. More studies should be done on developing high-quality subject searches, which calls for cooperation between medical subject experts and information specialists. In addition, there are limitations in using sensitivity and precision to evaluate the performances of information retrieval, which were discussed by Kagolovsky and Moehr [27].

## Conclusion

Since MEDLINE is the most widely used database in searching for evidence for systematic reviews, formulating an optimal search strategy to find RCTs in MEDLINE will greatly increase the efficiency of systematic reviews. Our study demonstrates that of the variants to HSSS_12 _proposed by Lefebvre and Clarke, adding the free text word *versus *to the first two phases of the HSSS provides a modest balance of the precision and sensitivity in the reviews studied here. We hope that this finding will become part of the evidence used by systematic reviewers and information specialists in making decisions on developing their search strategies for systematic reviews.

## Competing interests

The author(s) declare that they have no competing interests.

## Authors' contributions

LZ initially conceived of, designed, and executed the study, and wrote and revised the entire manuscript draft. IA reviewed the study design and results, and participated in the revision of the manuscript. MS participated in the study design, data extraction, drafting and revision of the manuscript. All authors read and approved the final manuscript.

**Table:** 

Appendix 1: Search strategies used	
Appendix 1: Search strategies used	
**Phase 1**	
RANDOMIZED CONTROLLED TRIAL.pt.	
CONTROLLED CLINICAL TRIAL.pt.	
RANDOMIZED CONTROLLED TRIALS.sh.	
RANDOM ALLOCATION.sh.	
DOUBLE BLIND METHOD.sh.	
SINGLE-BLIND METHOD.sh.	
or/1–6	
(ANIMAL not HUMAN).sh.	
7 not 8	
**Phase 2**	
RANDOMIZED CONTROLLED TRIAL.pt.	
CONTROLLED CLINICAL TRIAL.pt.	
RANDOMIZED CONTROLLED TRIALS.sh.	
RANDOM ALLOCATION.sh.	
DOUBLE BLIND METHOD.sh.	
SINGLE-BLIND METHOD.sh.	
or/1–6	
(ANIMAL not HUMAN).sh.	
18 or/10–17	
19 18 not 8	
20 19 not 9	
**Phase 3**	
21 COMPARATIVE STUDY.sh.	
22 exp EVALUATION STUDIES/	
23 FOLLOW UP STUDIES.sh.	
24 PROSPECTIVE STUDIES.sh.	
25 (control$ or prospectiv$ or volunteer$).ti, ab.	
26 or/21–25	
27 26 not 8	
28 27 not (9 or 20)	
29 9 or 20 or 28	
**Test Strategies**	
All 3 Phases	Subject SS and (9 or 20 or 28)
Top 2 Phases	Subject SS and (9 or 20)
Crossover	Subject SS and (9 or 20 or (crossover.ti, ab. not (ANIMAL not HUMAN).sh.))
CROSS-OVER STUDIES	Subject SS and (9 or 20 or (CROSS-OVER-STUDIES.sh. not (ANIMAL not HUMAN).sh.))
Volunteer	Subject SS and (9 or 20 or (volunteer.ti, ab. not (ANIMAL not HUMAN).sh.))
Versus	Subject SS and (9 or 20 or (versus.ti, ab. not (ANIMAL not HUMAN).sh.))

Subject SS is Subject search strategy, as presented in the review

**Table:** 

This known item search strategy was to identify included studies in Review 24, as listed in Appendix 3. There were two included studies in this review. Numbers in brackets were the number of records found in OVID MEDLINE.	
1 (Ekberg$ and Bjorkqvist$).au. and "1994" .yr. (3)	
2 from 1 keep 1 (1)	Line 1 and Line 2 identified 1^st ^included study
3 (Jensen$ and Nygren$).au. and "1995" .yr. (1)	Line 3 identified 2^nd ^included study
4 or/2–3 (2)	Line 4 pooled the two included studies by "or"

Subject SS is Subject search strategy, as presented in the review

## Pre-publication history

The pre-publication history for this paper can be accessed here:



## Supplementary Material

Additional file 1: Search strategies used Highly Sensitive Search Strategy (HSSS)

Additional file 2: Example known item search This known item search strategy was to identify included studies in Review 24, as listed in Additional file 3. There were two included studies in this review. Numbers in brackets were the number of records found in OVID MEDLINE.

Additional file 3: Systematic reviews of which search strategies were replicated
