# Supplementary material for: Optimizing search strategies to identify randomized controlled trials in MEDLINE
Source: BMC Med Res Methodol. 2006 May 9;6:23. doi: 10.1186/1471-2288-6-23 (PMC1488863; doi:10.1186/1471-2288-6-23)
Supplement: Additional file 1: Search strategies used — Highly Sensitive Search Strategy (HSSS) [file 1471-2288-6-23-S1.doc]

## Additional file 1: Search strategies used

Highly Sensitive Search Strategy (HSSS)

| Phase 1   1. RANDOMIZED CONTROLLED TRIAL.pt. 2. CONTROLLED CLINICAL TRIAL.pt. 3. RANDOMIZED CONTROLLED TRIALS.sh. 4. RANDOM ALLOCATION.sh. 5. DOUBLE BLIND METHOD.sh. 6. SINGLE-BLIND METHOD.sh. 7. or/1-6 8. (ANIMAL not HUMAN).sh. 9. 7 not 8 10. RANDOMIZED CONTROLLED TRIAL.pt. 11. CONTROLLED CLINICAL TRIAL.pt. 12. RANDOMIZED CONTROLLED TRIALS.sh. 13. RANDOM ALLOCATION.sh. 14. DOUBLE BLIND METHOD.sh. 15. SINGLE-BLIND METHOD.sh. 16. or/1-6 17. (ANIMAL not HUMAN).sh. | |
| --- | --- |
| Phase 2  10     CLINICAL TRIAL.pt.  11     exp CLINICAL TRIALS/  12     (clin$ adj25 trial$).ti,ab.  13     ((singl$ or doubl$ or trebl$ or tripl$) adj25 (blind$ or mask$)).ti,ab.  14     PLACEBOS.sh.  15     placebo$.ti,ab.  16     random$.ti,ab.  17     RESEARCH DESIGN.sh.  18     or/10-17  19     18 not 8  20     19 not 9 | |
| Phase 3  21 COMPARATIVE STUDY.sh.  22 exp EVALUATION STUDIES/  23 FOLLOW UP STUDIES.sh.  24 PROSPECTIVE STUDIES.sh.  25 (control$ or prospectiv$ or volunteer$).ti,ab.  26 or/21-25  27 26 not 8  28 27 not (9 or 20)  29 9 or 20 or 28 | |
| Test Strategies | |
| All 3 Phases | Subject SS and (9 or 20 or 28) |
| Top 2 Phases | Subject SS and (9 or 20) |
| Crossover | Subject SS and (9 or 20 or (crossover.ti,ab. not (ANIMAL not HUMAN).sh.)) |
| CROSS-OVER STUDIES | Subject SS and (9 or 20 or (CROSS-OVER-STUDIES.sh. not (ANIMAL not HUMAN).sh.)) |
| Volunteer | Subject SS and (9 or 20 or (volunteer.ti,ab. not (ANIMAL not HUMAN).sh.)) |
| Versus | Subject SS and (9 or 20 or (versus.ti,ab. not (ANIMAL not HUMAN).sh.)) |

Subject SS is Subject search strategy, as presented in the review
