# Supplementary material for: Optimizing search strategies to identify randomized controlled trials in MEDLINE
Source: BMC Med Res Methodol. 2006 May 9;6:23. doi: 10.1186/1471-2288-6-23 (PMC1488863; doi:10.1186/1471-2288-6-23)
Supplement: Additional file 2: Example known item search — This known item search strategy was to identify included studies in Review 24, as listed in Additional file 3. There were two included studies in this review. Numbers in brackets were the number of records found in OVID MEDLINE. [file 1471-2288-6-23-S2.doc]

## Additional file 2: Example known item search

This known item search strategy was to identify included studies in Review 24, as listed in Additional file 3. There were two included studies in this review. Numbers in brackets were the number of records found in OVID MEDLINE.

| 1 (Ekberg$ and Bjorkqvist$).au. and "1994".yr. (3) |  |
| --- | --- |
| 2 from 1 keep 1 (1) | Line 1 and Line 2 identified 1^st^ included study |
| 3 (Jensen$ and Nygren$).au. and "1995".yr. (1) | Line 3 identified 2^nd^ included study |
| 4 or/2-3 (2) | Line 4 pooled the two included studies by “or” |
