# Supplementary material for: Optimizing search strategies to identify randomized controlled trials in MEDLINE
Source: BMC Med Res Methodol. 2006 May 9;6:23. doi: 10.1186/1471-2288-6-23 (PMC1488863; doi:10.1186/1471-2288-6-23)
Supplement: Additional file 3: Systematic reviews of which search strategies were replicated [file 1471-2288-6-23-S3.doc]

## Additional file 3: Systematic reviews of which search strategies were replicated:

1. Athaullah N, Proctor M, Johnson NP: **Oral versus injectable ovulation induction agents for unexplained subfertility**. *Cochrane Database Syst Rev* 2002, **3**
2. Bell-Syer SEM, Hart R, Crawford F, Torgerson DJ, Tyrrell W, Russell I: **Oral treatments for fungal infections of the skin of the foot**. *Cochrane Database Syst Rev* 2002, **3**
3. Bonfill X, Serra C, Sacristan M, Nogue M, Losa F, Montesinos J: **Second-line chemotherapy for non-small cell lung cancer**. *Cochrane Database Syst Rev* 2002, **3**
4. Bowen A, Lincoln NB, Dewey M: **Cognitive rehabilitation for spatial neglect following stroke**. *Cochrane Database Syst Rev* 2002, **3**
5. Cody J, Daly C, Campbell M, Donaldson C, Grant A, Khan I: **Recombinant human erythropoietin for chronic renal failure anaemia in pre-dialysis patients**. *Cochrane Database Syst Rev* 2002, **3**
6. Crawford F, Hart R, Bell-Syer S, Torgerson D, Young P, Russell I: **Topical treatments for fungal infections of the skin and nails of the foot**. *Cochrane Database Syst Rev* 2002, **3**
7. Dickersin K, Manheimer E: **Surgery for nonarteritic anterior ischemic optic neuropathy**. *Cochrane Database Syst Rev* 2002, **3**
8. Dinh-Zarr T, DiGuiseppi C, Heitman E, Roberts I: **Interventions for preventing injuries in problem drinkers**. *Cochrane Database Syst Rev* 2002, **3**
9. Durkan A, Hodson EM, Willis NS, Craig JC: **Non-corticosteroid treatment for nephrotic syndrome in children**. *Cochrane Database Syst Rev* 2002, **3**
10. Esposito M, Worthington HV, Coulthard P, Jokstad A: **Interventions for replacing missing teeth: maintaining and re-establishing healthy tissues around dental implants**. *Cochrane Database Syst Rev* 2002, **3**
11. Evans JR: **Antioxidant vitamin and mineral supplements for age-related macular degeneration**. *Cochrane Database Syst Rev* 2002, **3**
12. Evans JR, Henshaw K: **Antioxidant vitamin and mineral supplementation for preventing age-related macular degeneration.** *Cochrane Database Syst Rev* 2002, **3**
13. Gibbs S, Harvey I, Sterling JC, Stark R: **Local treatments for cutaneous warts.** *Cochrane Database Syst Rev* 2002, **3**
14. Gibson JNA, Handoll HHG, Madhok R: **Interventions for treating proximal humeral fractures in adults**. *Cochrane Database Syst Rev* 2002, **3**
15. Gillespie LD, Gillespie WJ, Robertson MC, Lamb SE, Cumming RG, Rowe BH: **Interventions for preventing falls in elderly people**. *Cochrane Database Syst Rev* 2002, **3**
16. Hagen KB, Hilde G, Jamtvedt G, Winnem M: **Bed rest for acute low back pain and sciatica**. *Cochrane Database Syst Rev* 2002, **3**
17. Handoll HHG, Madhok R, Dodds C: **Anaesthesia for treating distal radial fracture in adults**. *Cochrane Database Syst Rev* 2002, **3**
18. Handoll HHG, Rowe BH, Quinn KM, de Bie R: **Interventions for preventing ankle ligament injuries**. *Cochrane Database Syst Rev* 2002, **3**
19. Handoll HHG, Madhok R, Howe TE: **Rehabilitation for distal radial fractures in adults**. *Cochrane Database Syst Rev* 2002, **3**
20. Handoll HHG, Madhok R: **Conservative interventions for treating distal radial fractures in adults**. *Cochrane Database Syst Rev* 2002, **3**
21. Handoll HHG, Madhok R: **Surgical interventions for treating distal radial fractures in adults**. *Cochrane Database Syst Rev* 2002, **3**
22. Hilde G, Hagen KB, Jamtvedt G, Winnem M: **Advice to stay active as a single treatment for low back pain and sciatica**. *Cochrane Database Syst Rev* 2002, **3**
23. Hodson EM, Knight JF, Willis NS, Craig JC: **Corticosteroid therapy for nephrotic syndrome in children**. *Cochrane Database Syst Rev* 2002, **3**
24. Karjalainen K, Hurri H, Jauhiainen M, Koes BW, Malmivaara A, Roine R: **Multidisciplinary rehabilitation for fibromyalgia and musculoskeletal pain in working age adults**. *Cochrane Database Syst Rev* 2002, **3**
25. Karjalainen K, Malmivaara A, van Tulder M, Roine R, Jauhiainen M, Hurri H: **Multidisciplinary biopsychosocial rehabilitation for neck and shoulder pain among working age adults.** *Cochrane Database Syst Rev* 2002, **3**
26. Karjalainen K, Malmivaara A, van Tulder M, Roine R, Jauhiainen M, Hurri H: **Multidisciplinary biopsychosocial rehabilitation for subacute low back pain among working age adults.** *Cochrane Database Syst Rev* 2002, **3**
27. Leyland M, Zinicola E: **Multifocal versus monofocal intraocular lenses after cataract extraction**. *Cochrane Database Syst Rev* 2002, **3**
28. Long V, Chen S: **Surgical interventions for bilateral congenital cataract**. *Cochrane Database Syst Rev* 2002, **3**
29. Lutters M, Vogt N: **Antibiotic duration for treating uncomplicated, symptomatic lower urinary tract infections in elderly women** *Cochrane Database Syst Rev* 2002, **3**
30. Mabey D, Fraser-Hurt N: **Antibiotics for trachoma**. *Cochrane Database Syst Rev* 2002, **3**
31. Macbeth F, Toy E, Coles B, Melville A, Eastwood A: **Palliative radiotherapy regimens for non-small cell lung cancer.** *Cochrane Database Syst Rev* 2002, **3**
32. MacLeod A, Daly C, Khan I, Vale L, Campbell M, Wallace S: **Cellulose, modified cellulose and synthetic membranes in the haemodialysis of patients with end-stage renal disease**. *Cochrane Database Syst Rev* 2002, **3**
33. Malthaner R, Fenlon D: **Preoperative chemotherapy for resectable thoracic esophageal cancer.** *Cochrane Database Syst Rev* 2002, **3**
34. Manyemba J, Mayosi BM: **Penicillin for secondary prevention of rheumatic fever**. *Cochrane Database Syst Rev* 2002, **3**
35. McLauchlan GJ, Handoll HHG: **Interventions for treating acute and chronic Achilles tendinitis**. *Cochrane Database Syst Rev* 2002, **3**
36. Moayyedi P, Soo S, Deeks J, Delaney B, Innes M, Forman D: **Pharmacological interventions for non-ulcer dyspepsia**. *Cochrane Database Syst Rev* 2002, **3**
37. Moayyedi P, Soo S, Delaney B, Harris A, Innes M, Oakes R: **Eradication of Helicobacter pylori for non-ulcer dyspepsia**. *Cochrane Database Syst Rev* 2002, **3**
38. Mulrow C, Lau J, Cornell J, Brand M: **Pharmacotherapy for hypertension in the elderly**. *Cochrane Database Syst Rev* 2002, **3**
39. Mulrow CD, Chiquette E, Angel L, Cornell J, Summerbell C, Anagnostelis B: **Dieting to reduce body weight for controlling hypertension in adults**. *Cochrane Database Syst Rev* 2002, **3**
40. Parker MJ, Gillespie LD, Gillespie WJ: **Hip protectors for preventing hip fractures in the elderly**. *Cochrane Database Syst Rev* 2002, **3**
41. Parkes J, Shepperd S: **Discharge planning from hospital to home**. *Cochrane Database Syst Rev* 2002, **3**
42. Pearce PK, Handoll HHG, Der Tavitian A: **Interventions for isolated diaphyseal fractures of the ulna in adults**. *Cochrane Database Syst Rev* 2002, **3**
43. Pratt BM, Woolfenden SR: **Interventions for preventing eating disorders in children and adolescents**. *Cochrane Database Syst Rev* 2002, **3**
44. Richter B, Neises G: **'Human' insulin versus animal insulin in people with diabetes mellitus**. *Cochrane Database Syst Rev* 2002, **3**
45. Sheikh A, Hurwitz B, Cave J: **Antibiotics versus placebo for acute bacterial conjunctivitis**. *Cochrane Database Syst Rev* 2002, **3**
46. Shelley MD, Barber J, Wilt T, Mason MD: **Surgery versus radiotherapy for muscle invasive** **bladder cancer**. *Cochrane Database Syst Rev* 2002, **3**
47. Shelley MD, Court JB, Kynaston H, Wilt TJ, Fish RG, Mason M: **Intravesical Bacillus Calmette-Guerin in Ta and T1 Bladder Cancer**. *Cochrane Database Syst Rev* 2002, **3**
48. Shepperd S, Iliffe S: **Hospital at home versus in-patient hospital care**. *Cochrane Database Syst Rev* 2002, **3**
49. Smeeth L, Iliffe S: **Community screening for visual impairment in the elderly** *Cochrane Database Syst Rev* 2002, **3**
50. Snellingen T, Evans JR, Ravilla T, Foster A: **Surgical interventions for age-related cataract**. *Cochrane Database Syst Rev* 2002, **3**
51. Snowden HM, Renfrew MJ, Woolrdige MW: **Treatments for breast engorgement during lactation**. *Cochrane Database Syst Rev* 2002, **3**
52. Srisurapanont M, Jarusuraisin N: **Opioid antagonists for alcohol dependence**. *Cochrane Database Syst Rev* 2002, **3**
53. Towheed T, Shea B, Wells G, Hochberg M: **Analgesia and non-aspirin, non-steroidal anti-inflammatory drugs for osteoarthritis of the hip**. *Cochrane Database Syst Rev* 2002, **3**
54. Van Perperstraten AM, Proctor ML, illipson G, ohnson NP: **Techniques for surgical retrieval of sperm prior to ICSI for azoospermia**. *Cochrane Database Syst Rev* 2002, **3**
55. Vimalachandra D, Craig JC, Cowell C, Knight JF: **Growth hormone for children with chronic renal failure**. *Cochrane Database Syst Rev* 2002, **3**
56. Wilkins M, Indar A, Wormald R: **Intra-operative Mitomycin C for glaucoma surgery**. *Cochrane Database Syst Rev* 2002, **3**
57. Wilt T, Ishani A, MacDonald R: **Serenoa repens for benign prostatic hyperplasia**. *Cochrane Database Syst Rev* 2002, **3**
58. Wilt T, MacDonald R, Ishani A, Rutks I, Stark G: **Cernilton for benign prostatic hyperplasia**. *Cochrane Database Syst Rev* 2002, **3**
59. Wilt T, Ishani A, MacDonald R, Stark G, Mulrow C, Lau J: **Beta-sitosterols for benign prostatic hyperplasia**. *Cochrane Database Syst Rev* 2002, **3**
60. Wormald R, Wilkins MR, Bunce C: **Post-operative 5-Fluorouracil for glaucoma surgery**. *Cochrane Database Syst Rev* 2002, **3**
61. Yeung EW, Yeung SS: **Interventions for preventing lower limb soft-tissue injuries in runners**. *Cochrane Database Syst Rev* 2002, **3**
